# Oral normality alterations and the impact on pneumonia, sepsis, and bloodstream infections in critically ill patients: a prospective cohort study

**DOI:** 10.62675/2965-2774.20260290

**Published:** 2026-06-24

**Authors:** Camila de Freitas Martins Soares Silveira, Celi Novaes Vieira, Alexandre Biasi Cavalcanti

**Affiliations:** 1 Universidade de São Paulo Faculdade de Medicina São Paulo SP Brazil Anesthesiology, Surgical Sciences, and Perioperative Medicine Program, Faculdade de Medicina, Universidade de São Paulo - São Paulo (SP), Brazil.; 2 Associação Brasileira de Odontologia Goiânia GO Brazil Associação Brasileira de Odontologia - Goiânia (GO), Brazil.; 3 HCor-Hospital do Coração Research Institute São Paulo SP Brazil Research Institute, HCor-Hospital do Coração - São Paulo (SP), Brazil.

**Keywords:** Healthcare-associated infection, Pneumonia, Bloodstream infection, Sepsis, Oral care, Critically illness

## Abstract

**Objective:**

To determine the prevalence of oral alterations among critical patients within the first 48 hours of intensive care unit admission, and their association with hospital-acquired pneumonia, bloodstream infections, and sepsis.

**Methods:**

This prospective cohort study was conducted from March 2018 to December 2021 in a Brazilian neurology intensive care unit. A single dental surgeon assessed oral conditions with follow-up until hospital discharge. Logistic regression analyzed the association between oral alterations and outcomes (hospital-acquired pneumonia, bloodstream infections, and sepsis), adjusting for confounders.

**Results:**

We enrolled 248 patients (55.6% male; mean age, 67.2 years), of whom 97.6% had oral abnormalities. The most common were visible dental plaque (61.7%), gingival inflammation (60.9%), and five or more missing teeth (49.2%). Carious teeth were linked to pneumonia (OR 1.10; 95%CI 1.00 - 1.20; p = 0.047). Destroyed teeth (OR 1.12; 95%CI 1.02 - 1.23; p = 0.02) and visible plaque (OR 1.08; 95%CI 1.00 - 1.17; p = 0.04) were associated with bloodstream infections. No factors were linked to sepsis.

**Conclusion:**

Most patients exhibited oral alterations upon admission to the intensive care unit. Carious teeth may increase pneumonia risk, while destroyed teeth and visible plaque may raise bloodstream infection risk.

## INTRODUCTION

Healthcare-associated infections (HAI) represent pathologies of high morbidity and mortality, impacting hospital costs, antimicrobial use, increased length of stay, and worsening patient experience.^(1)^ International organizations, as World Health Organization (WHO) and Centers for Disease Control and Prevention (CDC), and hospital internal committees conduct campaigns and preventive activities to minimize these adverse outcomes but pay little attention to oral characteristics and their relation with clinical outcomes, such as the evolution to hospital-acquired pneumonia, bloodstream infections, and sepsis.

Oral normality alterations associated with abnormalities such as residual roots, caries, and periodontal disease are commonly found in patients admitted to intensive care.^(2–5)^ During hospitalization, this clinical condition may deteriorate due to immunocompromise, lack of specific oral care, alteration in salivary flow, use of polypharmacy,^(6–9)^ oro-tracheal intubation, reduced level of consciousness, and lack of self-care.^(10)^ This increase in the load of potentially pathogenic microorganisms might predispose patients to infectious processes such as pneumonia and bloodstream infection.^(8,11–16)^

The prevalence of periodontal disease in intensive care unit (ICU) patients is 54.8% for gingivitis, 29.5% for periodontitis.^(4)^ In the case of a community disease the *Pesquisa Nacional de Saúde Bucal - SB Brasil* points to 28.4% of the population with periodontal disease of all manifestations.^(17)^

The oral health impairment in critically ill patients is not solely dependent on oral hygiene care, but is also caused by the worsening of pre-existing clinical conditions.^(4–6,10,11)^ A thorough diagnostic assessment and clinical dental interventions have proven effective in reducing hospital-acquired infections.^(4)^ The literature on the oral condition of ICU patients is limited; it suggests an association with pneumonia e and reinforces the need for further studies on the association with infectious diseases acquired during hospitalization.^(18,19)^ This study aimed to determine the prevalence of oral alterations among critical patients within the first 48 hours of ICU admission, and their association with hospital-acquired pneumonia, bloodstream infections, and sepsis.

## METHODS

### Ethical aspects and design

The study was approved by the Research Ethics Committee of the *Hospital das Clínicas* of *Faculdade de Medicina* of *Universidade de São Paulo* (USP) and by the Research Ethics Committee of the *Instituto de Neurologia de Goiânia*. Written informed consent was obtained from the study participants. We conducted a prospective cohort study with a non-consecutive convenience sample. A single dental surgeon evaluated the oral cavity of all enrolled patients within 48 hours after ICU admission. Follow-up was conducted until hospital discharge to determine clinical outcomes of pneumonia, bloodstream infections, and sepsis acquired during hospitalization.

### Characterization of the intensive care unit and hospital

The study was conducted from March 2018 to December 2021, in an adult ICU of a neurology-specialized hospital. The ICU had 20 beds, 10 for patients with post-operative or medical conditions with neurologic diseases (mostly stroke, dementia syndromes, Parkinson's, infectious neurological diseases) and 10 beds for patients with non-neurologic diseases.

Routine oral care consisted of toothbrushing with chlorhexidine for patients with teeth, and the use of a wooden spatula with gauze and chlorhexidine for edentulous patients.

### Patients

We included patients aged 18 years or older who were within the first 48 hours of ICU admission. We excluded patients with conditions preventing visual access to the oral cavity, such as facial or mouth lesions or conditions that precluded evaluation within 48 hours of ICU admission. Patients with a clear indication for emergency dental care, such as severe abscesses, as determined by the dental surgeon and physician, were also excluded.

### Sample size

The sample size was defined by convenience. With approximately 250 patients, the study can estimate the prevalence of oral alterations with 95% confidence intervals (95%CI) and a maximum width of 12.5%. To assess the association between oral alterations and the outcomes of hospital-acquired pneumonia, bloodstream infection, and sepsis, we expected 10 to 20% event rates (approximately 25 to 50 cases) within this sample size. With this number of events, it is suggested that a maximum of two to five independent variables be included in the logistic regression models.^(20)^

### Data collection and management

We collected demographic and laboratory data, as well as systemic conditions (underlying diseases and reason for hospitalization), from medical records, and the prognostic scores Simplified Acute Physiology Score (SAPS) 3 and Sequential Organ Failure Assessment (SOFA) from the Epimed Monitor system. Information on the evolution of HAI was collected from the hospital infection control service database, and patients were followed up until discharge.

A single dental surgeon with extensive experience in hospital dentistry conducted all oral assessments non-invasively. Dental status was assessed by the presence of clinically visible carious lesions on teeth in the occlusal, lingual, vestibular, incisal, cervical, and interproximal regions, without the need for radiographic images.

The following definitions were used: destroyed teeth - coronal destruction involving four or more tooth surfaces; missing teeth - teeth that were absent at the time of assessment and had been removed prior to hospitalization; fungal infections - presence of an erythematous or pseudomembranous region on the mucosa, with or without symptoms and responsive to antifungal therapy; bacterial infections (abscess or fistula): regions associated with a visible increase in intraoral or extraoral volume, with or without the presence of fistulas in the buccal or lingual alveolar ridge region were considered. We examined the lingual dorsum for the presence of visible lingual biofilm and visible papillae. We classified cases with visible biofilm into present on one third of the lingual dorsum (corresponding to only the posterior third), two thirds of the lingual dorsum (posterior and middle thirds), and three thirds of the lingual dorsum (posterior, middle, and anterior thirds). We considered papillae as not visible when lingual papillae were covered by lingual biofilm or had been mechanically removed. Salivary status was assessed by visual organoleptic analysis: the presence or absence of saliva (asialia) in the oral cavity and/or oropharynx; salivary viscosity, by bidigital analysis of the residual saliva present in the oral environment, considering it as fluid saliva if the salivary sample collected between the observer's index and thumb fingers does not form a salivary filament or breaks easily, and viscous saliva if the salivary filament formed is in strands of 2 to 4cm or more measured on a disposable ruler; the presence or absence of sialostasis in the oropharynx, represented by saliva dammed up in the oropharyngeal region, visible clinically.^(21,22)^

Periodontal indices were assessed by visible dental plaque on the buccal surface and signs of marginal gingival inflammation, through spontaneous bleeding or after digital compression along the alveolar ridge, in the occlusal peak direction. We did not perform systematic periodontal probing to avoid the risk of bacteremia associated with this invasive procedure. As for the presence of mucosal lesions, we considered visible traumatic lesions on the lips, tongue, and alveolar ridge; mucositis induced by chemotherapy or pharmacodermia; pressure lesions from devices and manifestations suggestive of viral infections, but due to limitations of the local service, it was not possible to carry out complementary laboratory tests.

### Outcomes

The outcomes of interest were pneumonia, sepsis, and bloodstream infection acquired during hospitalization, occurring from baseline data collection after study enrollment until hospital discharge. The hospital infection control service diagnosed hospital-acquired pneumonia and bloodstream infection according to the definitions of the *Agência Nacional de Vigilância Sanitária* (ANVISA),^(23,24)^ and the diagnosis of sepsis according to the definition of the *Instituto Latino-Americano de Sepse* (ILAS).^(25)^ We also determine the length of stay and mortality in the ICU and in the hospital.

### Statistical analysis

Categorical variables are presented as absolute frequencies and percentages. Continuous variables are presented as mean and standard deviation (SD) or as median and interquartile range, depending on whether the distribution of the variable is consistent with normality. The distribution of variables was assessed visually using frequency histograms. 95%CI are presented for the prevalence of oral alterations.

Logistic regression was used to assess the association between each change in oral normality and the outcomes hospital-acquired pneumonia, bloodstream infection, and sepsis. The logistic regression models were adjusted for baseline variables selected a priori that could be confounders, i.e., could cause both the oral alterations and the outcomes of interest. The baseline variables included in the logistic regression models for hospital-acquired pneumonia and sepsis were comorbidity, corticosteroid use in the last 3 months, dysphagia, tracheal intubation, and age. For hospital-acquired bloodstream infection, the adjusted variables were comorbidity, corticosteroid use in the last 3 months, and age. The results presented are adjusted odds ratios (OR), 95%CIs, and p values.

A significance level of 0.05 was used, and no adjustment for multiple comparisons was made. Therefore, all results should be interpreted as exploratory. The analyses were carried out using R software version 4.03 (R Foundation for Statistical Computing).

## RESULTS

### Patients

A total of 261 patients were assessed for eligibility; 12 were excluded due to severe abscesses requiring immediate surgery, and 1 was excluded because the patient was under 18 years of age. Of the 248 patients included, 55.6% were male, the average age was 67.2 years, and the median SAPS 3 was 55 (Table 1). One quarter of patients received oral hygiene from their caregivers. Most patients were admitted for medical problems. The most common were cardiovascular, respiratory, and neurological diseases. The most common associated comorbidities were diabetes mellitus, neoplasms, rheumatological pathologies, and chronic kidney disease.

**Table 1:** Characteristics of the included patients*

Basic characteristics		
Age (years)		72 (57.5 - 80)
Female gender		110 (44.4)
SAPS 3		55 (45 - 62)
SOFA		4 (3 - 6)
Type of admission		
	Clinic	165 (66.5)
	Elective surgery	34 (13.7)
	Emergency surgery	49 (19.8)
Reason for hospitalization*		
	Respiratory disease	100 (40.3)
	Neurological disease	79 (31.8)
	COVID-19	5 (2.0)
	Cardiovascular reasons for hospitalization	168 (67.7)
	Other causes	0,0
Comorbidities		
	Neoplasms	41 (16.5)
	Human immunodeficiency virus	3 (1.2)
	Previous solid transplant	1 (0.4)
	Rheumatological diseases	29 (11.7)
	Chronic kidney disease (creatinine ≥ 2mg/dL)	28 (11.3)
	Diabetes	64 (25.8)
Homecare patient		61 (24.6)
Responsible for oral hygiene		
	Patient	185 (74.6)
	Caregiver	63 (25.4)
Dysphagia		161 (64.9)
Smoking		
	Current	41 (16.5)
	Former smoker	46 (18.6)
Corticosteroids in the last 3 months		68 (27.4)
Orotracheal intubation		93 (37.5)

SAPS 3 - Simplified Acute Physiology Score 3; SOFA - Sequential Organ Failure Assessment. Chronic kidney disease is defined as a creatinine ≥ 2mg/dL.

* Each patient could have more than one reason for being admitted to the intensive care unit. Results expressed as n (%) or median (interquartile range).

### Clinical outcomes

Sepsis occurred in 76 (30.6%) cases; 31 patients (12.5%) developed hospital-acquired pneumonia; and hospital-acquired bloodstream infection occurred in 26 (10.5%) cases. The average ICU stay was 16.8 days, and the average hospital stay was 24.7 days, with ICU mortality of 21.8% and hospital mortality of 25%.

### Prevalence of changes in oral normality

Prevalence of changes in oral normality are described in table 2. A total of 242 patients (95%CI 94.6 to 99.0) had at least one of the 17 oral normality alterations analyzed. Several oral normality alterations were present in at least 25% of the patients: presence of decayed teeth (36%), visible dental plaque (61,7%), gum inflammation (60,9%), asialia (18,5), or viscous saliva (41,9%) sialostasis in the oropharynx (43,1%), mucosal lesions (38,7%), and papillae not visible (27,4%). Destroyed teeth and signs of fungal infection were alterations found in more than 20% of patients.

**Table 2:** Prevalence of oral normality alterations*

Variable		All patients (n = 248)	
		n	% (95%CI)
Any oral abnormality		242	97.6 (94.6 - 99.0)
Decayed teeth (≥ 1)†		89	36.0 (30.1 - 42.4)
Destroyed teeth (≥ 1)‡		52	21.1 (16.2 - 26.8)
Fungal infection§		52	21.0 (16.2 - 26.7)
Bacterial infections (abscesses or fistulas)¶		19	7.7 (4.8 - 11.9)
Visible lingual biofilm			
	Absent	91	36.7 (30.7 - 43.1)
	Present on 1/3 of the lingual dorsum	40	16.1 (11.9 - 21.4)
	Present on 2/3 of the lingual dorsum	71	28.6 (23.2 - 34.8)
	Present on 3/3 of the lingual dorsum	46	18.5 (14.0 - 24.1)
Visible dental plaque||		153	61.7 (55.3 - 67.7)
Gum inflammation#		151	60.9 (54.5 - 66.9)
Salivary status**			
	Fluid	98	39.5 (33.4 - 45.9)
	Viscous	104	41.9 (35.8 - 48.4)
	Asialia	46	18.5 (14.0 - 24.1)
Sialostasis in the oropharynx		107	43.1 (36.9 - 49.6)
Missing teeth (6 or more)		122	49.2 (42.8 - 55.6)
Mucosal lesions††		96	38.7 (32.7 - 45.1)
Papillae not visible		68	27.4 (22.1 - 33.5)

* The data presented are numerator, percentage and confidence interval;

† decayed teeth are assessed through the presence of carious lesions on teeth in the occlusal, lingual, buccal, incisal, cervical and interproximal regions that are clinically visible, without the need for radiographic imaging;

‡ destroyed teeth present coronal destruction of four or more tooth surfaces;

§ fungal infection was diagnosed by observing pseudomembranes on soft tissues (pseudomembranous candida) or erythematous areas, both of which responded to antifungal therapy;

¶ bacterial infections: were considered to be those associated with a visible increase in intra- or extraoral volume with or without the presence of fistulas in the region of the alveolar ridge on the buccal and/or lingual side;

|| dental plaque visible on the buccal side of the teeth;

# gingival inflammation defined by the presence of spontaneous gingival bleeding or after digital compression along the alveolar ridge in the occlusal or incisal apex direction;

** salivary status (fluid or viscous) is assessed through a salivary sample contained between the index and thumb fingers, measured with an endodontic ruler;

†† mucosal lesions were considered to be visible traumatic lesions on the lips, tongue and alveolar ridge, mucositis induced by chemotherapy or pharmacodermia on all intraoral mucous membranes and lips, pressure lesions from devices and manifestations suggestive of viral infections.

### Association between changes in oral normality and clinical outcomes

Among the 31 patients who developed hospital-acquired pneumonia, the 17 variables collected during the oral cavity assessment, decayed teeth showed a statistically significant independent association with pneumonia. Seventeen (54.8%) had decayed teeth, compared to 72 (33.3%) among the 216 patients without pneumonia (OR 1.10; 95%CI 1.00 to 1.20; p = 0.04) shown in table 3.

**Table 3:** Association between oral normality alterations and hospital-acquired pneumonia* adjusted for comorbidities†, corticoid use, dysphagia, orotracheal intubation and age

Explanatory variables		Total n/N (%)	Pneumonia n/N (%)		Estimate (95%CI)	p value
			Yes	No		
Decayed teeth‡		89/247 (36.0)	17/31 (54.8)	72/216 (33.3)	1.10 (1.00 - 1.20)	0.047
Destroyed teeth§		52/247 (21.1)	6/31 (19.4)	46/216 (21.3)	0.95 (0.85 - 1.07)	0.40
Fungal infection¶		52/248 (21.0)	7/31 (22.6)	45/217 (20.7)	1.01 (0.90 - 1.13)	0.87
Bacterial infection||		19/248 (7.7)	2/31 (6.5)	17/217 (7.8)	0.95 (0.80 - 1.13)	0.58
Visible lingual biofilm						
	0	91/248 (36.7)	12/31 (38.7)	79/217 (36.4)	1 (reference)	
	1 *versus* 0	40/248 (16.1)	6/31 (19.4)	34/217 (15.7)	1.06 (0.93 - 1.21)	0.36
	2 *versus* 0	71/248 (28.6)	8/31 (25.8)	63/217 (29.0)	1.01 (0.90 - 1.13)	0.85
	3 *versus* 0	46/248 (18.5)	5/31 (16.1)	41/217 (18.9)	0.99 (0.87 - 1.13)	0.87
Visible dental plaque#		153/248 (61.7)	18/31 (58.1)	135/217 (62.2)	0.98 (0.90 - 1.08)	0.72
Gum inflammation**		151/248 (60.9)	20/31 (64.5)	131/217 (60.4)	1.03 (0.94 - 1.13)	0.47
Saliva††						
	Fluid	98/248 (39.5)	13/31 (41.9)	85/217 (39.2)	1 (ref)	
	Viscous *versus* fluid	104/248 (41.9)	12/31 (38.7)	92/217 (42.4)	0.98 (0.89 - 1.09)	0.75
	No saliva *versus* fluid	46/248 (18.5)	6/31 (19.4)	40/217 (18.4)	0.97 (0.85 - 1.10)	0.63
Sialostasis in the oropharynx		107/248 (43.1)	15/31 (48.4)	92/217 (42.4)	1.07 (0.96 - 1.18)	0.23
Missing teeth (6 or more)		122/248 (49.2)	11/31 (35.5)	111/217 (51.2)	0.96 (0.87 - 1.07)	0.47
Mucosal lesion‡‡		96/248 (38.7)	13/31 (41.9)	83/217 (38.2)	1.04 (0.95 - 1.14)	0.42
Papillae not visible		68/248 (27.4)	11/31 (35.5)	57/217 (26.3)	1.08 (0.98 - 1.19)	0.14

* Period of hospitalization corresponds to the period until hospital discharge regardless of vital status. The numbers presented under evolution to pneumonia, yes or no, are the numerators (percentages). Results (*odds ratio*, 95%CI, and p) for each oral abnormality obtained from logistic regression models adjusted for: comorbidity, corticosteroid use, dysphagia, intubation, and age;

† comorbidities are diabetes, chronic kidney disease, heart failure, AIDS, previous transplant, and rheumatological disease. Confidence interval.

‡ decayed teeth are assessed through the presence of carious lesions on teeth in the occlusal, lingual, buccal, incisal, cervical and interproximal regions that are clinically visible, without the need for radiographic imaging;

§ destroyed teeth present coronal destruction of four or more tooth surfaces;

¶ fungal infection was diagnosed by observing pseudomembranes on soft tissues (*pseudomembranous candida*) or erythematous areas, both of which responded to antifungal therapy;

|| bacterial infections: were considered to be those associated with a visible increase in intra- or extraoral volume with or without the presence of fistulas in the region of the alveolar ridge on the buccal and/or lingual side;

# dental plaque visible on the buccal side of the teeth;

** gingival inflammation defined by the presence of spontaneous gingival bleeding or after digital compression along the alveolar ridge in the occlusal or incisal apex direction;

†† salivary status (fluid or viscous) is assessed through a salivary sample contained between the index and thumb fingers, measured with an endodontic ruler;

‡‡ mucosal lesions were considered to be visible traumatic lesions on the lips, tongue and alveolar ridge, mucositis induced by chemotherapy or pharmacodermia on all intraoral mucous membranes and lips, pressure lesions from devices and manifestations suggestive of viral infections.

The changes in oral normality that showed a statistically significant independent association with bloodstream infection were the presence of destroyed teeth (OR 1.12; 95%CI 1.02 to 1.23; p = 0.02) and visible plaque (OR 1.08; 95%CI 1.0 to 1.17; p = 0.04) (Table 4). None of the variables collected during the evaluation of the oral cavity showed a statistically significant independent association with sepsis (Table 5).

**Table 4:** Association between oral normality alterations and bloodstream infection acquired during hospitalization*. Models for Bloodstream Infection adjusted for comorbidities†, corticosteroid use and age

Explanatory variables		Total n/N (%)	Blood infection n/N (%)		Estimate (95%CI)	p value
			Yes	No		
Decayed teeth‡		89/247 (36.0)	9/26 (34.6)	80/222 (36.2)	0.99 (0.92 - 1.08)	0.87
Destroyed teeth§		52/247 (21.1)	10/26 (38.5)	42/222 (19.0)	1.12 (1.02 - 1.23)	0.02
Fungal infection¶		52/248 (21.0)	6/26 (23.1)	46/222 (20.7)	1.00 (0.91 - 1.10)	0.94
Bacterial infection||		19/248 (7.7)	4/26 (15.4)	15/222 (6.8)	1.12 (0.97 - 1.29)	0.13
Visible lingual biofilm						
	0	91/248 (36.7)	11/26 (42.3)	80/222 (36.0)	1 (ref)	-
	1 *versus* 0	40/248 (16.1)	4/26 (15.4)	36/222 (16.2)	0.97 (0.87 - 1.09)	0.61
	2 *versus* 0	71/248 (28.6)	8/26 (30.8)	63/222 (28.4)	0.99 (0.90 - 1.09)	0.81
	3 *versus* 0	46/248 (18.5)	3/26 (11.5)	43/222 (19.4)	0.94 (0.85 - 1.05)	0.30
Visible dental plaque#		153/248 (61.7)	21/26 (80.8)	132/222 (59.5)	1.08 (1.00 - 1.17)	0.04
Gum inflammation**		151/248 (60.9)	19/26 (73.1)	132/222 (59.5)	1.05 (0.98 - 1.14)	0.18
Saliva††						
	Fluid	98/248 (39.5)	9/26 (34.6)	89/222 (40.1)	1 (ref)	-
	Viscous *versus* fluid	104/248 (41.9)	12/26 (46.2)	92/222 (41.4)	1.02 (0.93 - 1.11)	0.68
	No saliva *versus* fluid	46/248 (18.5)	5/26 (19.2)	41/222 (18.5)	1.01 (0.90 - 1.12)	0.88
Sialostasis in the oropharynx		107/248 (43.1)	12/26 (46.2)	95/222 (42.8)	1.02 (0.94 - 1.10)	0.63
Missing teeth (6 or more)		122/248 (49.2)	13/26 (50.0)	109/222 (49.1)	1.00 (0.92 - 1.09)	0.97
Mucosal lesion‡‡		96/248 (38.7)	12/26 (46.2)	84/222 (37.8)	1.02 (0.94 - 1.11)	0.59
Papillae not visible		68/248 (27.4)	9/26 (34.6)	59/222 (26.6)	1.04 (0.95 - 1.13)	0.39

* Period of hospitalization corresponds to the period until hospital discharge regardless of vital status. The numbers presented under evolution to bloodstream infection, yes or no, are the numerators (percentages). Results (odds ratio, 95% confidence interval and p value) for each oral abnormality obtained from logistic regression models adjusted for

† comorbidities (diabetes, chronic kidney disease, heart failure, AIDS, previous transplant and rheumatologic disease), corticoid use and age (denominator bloodstream infection category no = 222);;

‡ decayed teeth are assessed through the presence of carious lesions on teeth in the occlusal, lingual, buccal, incisal, cervical and interproximal regions that are clinically visible, without the need for radiographic imaging

§ destroyed teeth present coronal destruction of four or more tooth surfaces;

¶ fungal infection was diagnosed by observing pseudomembranes on soft tissues (*pseudomembranous candida*) or erythematous areas, both of which responded to antifungal therapy;

|| bacterial infections: were considered to be those associated with a visible increase in intra- or extraoral volume with or without the presence of fistulas in the region of the alveolar ridge on the buccal and/or lingual side;

# dental plaque visible on the buccal side of the teeth;

** gingival inflammation defined by the presence of spontaneous gingival bleeding or after digital compression along the alveolar ridge in the occlusal or incisal apex direction;

†† salivary status (fluid or viscous) is assessed through a salivary sample contained between the index and thumb fingers, measured with an endodontic ruler;

‡‡ mucosal lesions were considered to be visible traumatic lesions on the lips, tongue and alveolar ridge, mucositis induced by chemotherapy or pharmacodermia on all intraoral mucous membranes and lips, pressure lesions from devices and manifestations suggestive of viral infections. 95%CI - 95% confidence interval

**Table 5:** Association between oral normality alterations and hospital-acquired sepsis*, sepsis models adjusted for comorbidity†, corticosteroid use, dysphagia, tracheal intubation and age‡

Explanatory variables		Total n/N (%)	Sepsis n/N (%)		Estimate (95%CI)	p value
			Yes	No		
Decayed teeth§		89/247 (36.0)	22/76 (28.9)	67/172 (39.2)	0.91 (0.80 - 1.02)	0.11
Destroyed teeth¶		52/247 (21.1)	13/76 (17.1)	39/172 (22.8)	0.93 (0.81 - 1.07)	0.34
Fungal infection||		52/248 (21.0)	22/76 (28.9)	30/172 (17.4)	1.12 (0.97 - 1.29)	0.12
Bacterial infection#		19/248 (7.7)	7/76 (9.2)	12/172 (7.0)	1.06 (0.85 - 1.31)	0.60
Visible lingual biofilm						
	0	91/248 (36.7)	30/76 (39.5)	61/172 (35.5)	1 (ref)	-
	1 *versus* 0	40/248 (16.1)	9/76 (11.8)	31/172 (18.0)	0.88 (0.74 - 1.04)	0.13
	2 *versus* 0	71/248 (28.6)	20/76 (26.3)	51/172 (29.7)	0.94 (0.81 - 1.08)	0.39
	3 *versus* 0	46/248 (18.5)	17/76 (22.4)	29/172 (16.9)	1.05 (0.89 - 1.23)	0.59
Visible dental plaque**		153/248 (61.7)	45/76 (59.2)	108/172 (62.8)	0.96 (0.85 - 1.08)	0.48
Gum inflammation††		151/248 (60.9)	42/76 (55.3)	109/172 (63.4)	0.93 (0.82 - 1.04)	0.20
Saliva‡‡						
	Fluid	98/248 (39.5)	26/76 (34.2)	72/172 (41.9)	1 (ref)	-
	Viscous *versus* fluid	104/248 (41.9)	33/76 (43.4)	71/172 (41.3)	1.04 (0.92 - 1.18)	0.53
	No saliva *versus* fluid	46/248 (18.5)	17/76 (22.4)	29/172 (16.9)	1.11 (0.94 - 1.31)	0.23
Sialostasis in the oropharynx		107/248 (43.1)	36/76 (47.4)	71/172 (41.3)	1.03 (0.91 - 1.18)	0.61
Missing teeth (6 or more)		122/248 (49.2)	39/76 (51.3)	83/172 (48.3)	0.96 (0.84 - 1.10)	0.56
Mucosal lesion§§		96/248 (38.7)	35/76 (46.1)	61/172 (35.5)	1.06 (0.94 - 1.20)	0.37
Papillae not visible		68/248 (27.4)	18/76 (23.7)	50/172 (29.1)	0.93 (0.82 - 1.06)	0.28

* Period of hospitalization corresponds to the period until hospital discharge regardless of vital status. The numbers presented under evolution to sepsis, yes or no, are the numerators (percentages). Results (*odds ratio*, 95% confidence interval and p value) for each oral abnormality obtained from logistic regression models adjusted for

† comorbidities (diabetes, chronic kidney disease, heart failure, AIDS, previous transplant and rheumatologic disease), corticoid use and age (denominator sepsis = 172);

§ decayed teeth are assessed through the presence of carious lesions on teeth in the occlusal, lingual, buccal, incisal, cervical and interproximal regions that are clinically visible, without the need for radiographic imaging

¶ destroyed teeth present coronal destruction of four or more tooth surfaces;

|| fungal infection was diagnosed by observing pseudomembranes on soft tissues (*pseudomembranous candida*) or erythematous areas, both of which responded to antifungal therapy;

# bacterial infections: were considered to be those associated with a visible increase in intra- or extraoral volume with or without the presence of fistulas in the region of the alveolar ridge on the buccal and/or lingual side;

** dental plaque visible on the buccal side of the teeth;

†† gingival inflammation defined by the presence of spontaneous gingival bleeding or after digital compression along the alveolar ridge in the occlusal or incisal apex direction;

‡‡ salivary status (fluid or viscous) is assessed through a salivary sample contained between the index and thumb fingers, measured with an endodontic ruler;

§§ mucosal lesions were considered to be visible traumatic lesions on the lips, tongue and alveolar ridge, mucositis induced by chemotherapy or pharmacodermia on all intraoral mucous membranes and lips, pressure lesions from devices and manifestations suggestive of viral infections. 95%CI - 95% confidence interval.

## DISCUSSION

Among the 17 alterations in oral normality assessed, decayed teeth may increase the risk of hospital-acquired pneumonia during hospitalization. Destroyed teeth and visible plaque may increase the risk of hospital-acquired bloodstream infection. None of the changes in oral normality were associated with hospital-acquired sepsis.

The carious lesions associated with the development of pneumonia are pre-existing, since the sample was collected within 48 hours of ICU admission. Studies show that pre-existing oral health is a specific risk factor in the development of ventilator-associated pneumonia.^(26)^ Microorganisms appear to colonize primarily hard tissues (teeth) and then soft tissues (gums and mucous membranes) and may already migrate to the lower airways before infecting other oral tissues.^(27)^ The primary route of pulmonary contamination is bronchoaspiration of pulmonary^(8)^ pathogens, but recently the hematogenous route has also been considered. Infections in the periapex, originating from teeth destroyed by caries such as those included in this sample, migrate to the cervical, thoracic, and pulmonary vascularization, forming an oral-vasculo-pulmonary pathway.^(14,16,28)^

Given that the periapical region is extensively vascularized and that pathogens from cavities in teeth destroyed by caries survive in this periapical region without saliva access or the ability to exert an immune response, having free access to the vascular plexus,^(14,16)^ it is suggested that this is a possible route for association with bloodstream infection. Visible plaque, also associated with this healthcare-associated infection, understood within the scope of periodontal indices evaluated in this study, resulting from the heterogeneous accumulation of aerobic and anaerobic bacteria that form an organized biofilm adherent to the surface of the teeth and periodontal tissue^(29,30)^ presents the possibility of hematogenous dissemination through the vessels present in the periodontal region with the risk of developing a bloodstream infection.^(12–14,31,32)^ These findings corroborate the clinical relevance of controlling oral microorganisms for the prevention of bloodstream infections.^(33)^

Although 76 patients developed hospital-acquired sepsis, no oral alterations were associated with the problem. Asymptomatic necrosis is common, diagnosis is late, and, when there is no intervention, it can evolve into severe odontogenic infection and sepsis.^(34,35)^ In our study, the sequence of severe odontogenic abscesses occurred in 12 patients who, due to clinical and dental medical imperatives, required immediate intervention and were excluded from the analysis. If they were kept, they had a potential risk of developing into sepsis, as suggested in the literature.

Among patients admitted to the ICU for up to 2 days, almost all presented with one or more alterations in oral normality, with a predominance of decayed teeth, visible dental plaque, gum inflammation, asialia or viscous saliva, sialostasis in the oropharynx, mucosal lesions, and papillae not visible.

The prevalence of oral normality alterations found in this study is similar to that found in other Brazilian ICU by Bellissimo-Rodrigues et al.,^(4)^ in which of the 254 ICU patients assessed, the highest prevalence was periodontal disease (54.8% had gingivitis, 29.5% periodontitis), decayed teeth (29.1%), and destroyed teeth with residual roots (29.1%).^(3)^ On the other hand, the oral health of critically ill patients is worse compared to that of community patients. For example, the Brazil Oral Health Program reports 28.4% of the population with periodontal disease of all manifestations,^(17)^ whereas in our study we observed 60.9% involvement of tooth-supporting tissues. This clinical periodontal deterioration may be associated with ineffective oral hygiene, antibiotic or immunosuppressant use, an open oral cavity, altered salivary flow, and a hospital environment, all of which significantly alter its composition, resulting in an increase in the quantity and complexity of this biofilm.^(7,36–38)^ In addition, poor periodontal health might be caused by oral hygiene provided by caregivers in one-fourth of the patients.

Contrasting the data from this study with the survey of Brazilian ICUs conducted by the *Associação de Medicina Intensiva Brasileira* (AMIB), which assessed age, gender, and comorbidities, we observed the same age group and similar data on chronic kidney disease and neoplastic diseases. Similar values for hospitalization for diabetes and a higher number in our study of patients admitted for cardiac.

Our study has strengths. A single dentist experienced in critically ill dentistry conducted all assessments prospectively over a period of 2 years and 9 months. While studies on oral conditions *versus* systemic outcomes mostly cover only periodontal disease, caries, and decayed teeth, we assessed 17 alterations of oral normality. We also assessed a proper salivary pattern, which is essential to achieve a balanced microbiota and prevent fungal infections.^(39,40)^

The study has limitations. It was conducted in a single center in Brazil. The prevalence of oral conditions may differ in other regions. We did not carry out periodontal probing. It is possible that if we had carried out this subgingival assessment, we would have diagnosed more patients with periodontal disease. Conversely, as it is an invasive procedure, we might have increased the risk of microorganism penetration and bacteremia.^(41–43)^ Infections device-associated significantly contribute to hospital mortality and impose a high excess risk of death for critically ill patients.^(44)^ The definitions of pneumonia and bloodstream infection were followed by the ANVISA criteria, and sepsis was defined by the ILAS criteria, according to the Hospital Infection Control Commission. This fact may limit the replication of this study in units outside the Brazilian territory. Residual confounding is another potential limitation, as in any observational study. In our study, we aimed to control for confounding by adjusting for a limited number of potential confounders. Other variables we did not adjust for may also be confounders.

Other variables may also be associated with both oral normality alterations present within the first hours after ICU admission and the outcomes of interest.

## CONCLUSION

Almost all critically ill patients had oral abnormalities present within the first 48 hours after intensive care unit admission, including visible dental plaque, gingival inflammation, decayed teeth, and loss of five or more teeth. The presence of carious teeth was associated with increased risk of hospital-acquired pneumonia, while destroyed teeth and visible dental plaque were associated with increased risk of hospital-acquired bloodstream infections. No oral abnormality was associated with the incidence of hospital-acquired sepsis. Further studies are needed to assess the effectiveness of dental interventions in preventing healthcare-associated infections among critically ill patients.

## Data Availability

The contents are already available.
